# Cocaine and Alcohol Co-Ingestion-Induced Severe Rhabdomyolysis With Acute Kidney Injury Culminating in Hemodialysis-Dependent End-Stage Renal Disease: A Case Report and Literature Review

**DOI:** 10.7759/cureus.8595

**Published:** 2020-06-13

**Authors:** Sasmit Roy, Venu Madhav Konala, Sreedhar Adapa, Srikanth Naramala, Subhasish Bose

**Affiliations:** 1 Nephrology, University of Virginia, Lynchburg, USA; 2 Nephrology, Liberty University Medical School, Lynchburg, USA; 3 Hematology and Oncology, Ashland Bellefonte Cancer Center, Ashland, USA; 4 Hematology and Oncology, King's Daughters Medical Center, Ashland, USA; 5 Nephrology, Kaweah Delta Medical Center, Visalia, USA; 6 Rheumatology, Adventist Medical Center, Hanford, USA; 7 Nephrology / Internal Medicine, Lynchburg General Hospital, Lynchburg, USA; 8 Nephrology, Liberty University College of Osteopathic Medicine, Lynchburg, USA; 9 Nephrology, University of Virginia, Charlottesville, USA

**Keywords:** cocaine ingestion, cocaethylene, rhabdomyolysis, acute kidney injury, hemodialysis

## Abstract

Cocaine toxicity is associated with several organ dysfunctions, including acute kidney injury (AKI). Rhabdomyolysis is the most likely mechanism that mediates AKI, and associated alcohol co-ingestion can amplify the situation. AKI, if severe, can result in end-stage renal disease (ESRD) requiring renal replacement therapy (RRT). All patients with cocaine intoxication should be screened for rhabdomyolysis and AKI along with testing for other drug toxicity, including alcohol. Aggressive measures should be taken to treat the underlying cause that contributes to AKI, and the patients need to be educated about this severe condition. Our patient is a unique case where cocaine and alcohol co-ingestion led to severe rhabdomyolysis, AKI, and subsequently developed ESRD requiring ongoing hemodialysis (HD). He was on daily cocaine and alcohol co-ingestion for seven days and subsequently developed AKI with oliguria from rhabdomyolysis. His creatine kinase (CK) was significantly elevated to 141974 IU/L, and his serum creatinine was 11 mg/dl. Despite aggressive intravenous hydration, his kidney function did not improve, and he ended up needing HD for more than one year despite abstaining from cocaine.

## Introduction

Cocaine intoxication is very commonly associated with emergency room visits in the United States (US) [1]. Cocaine toxicity can cause various organ dysfunction, including severe rhabdomyolysis. Acute kidney injury (AKI) from heme-induced renal tubular damage is a dreadful complication of cocaine-induced rhabdomyolysis [2]. Alcohol co-ingestion compounds this effect as it can itself lead to profound rhabdomyolysis and tends to potentiate cocaine toxicity [3]. Severe AKI may often need renal replacement therapy (RRT). No case of end-stage renal disease (ESRD) or prolonged dialysis requirement from cocaine and alcohol co-ingestion leading to rhabdomyolysis has been reported so far. Our case highlights this dreaded occurrence in this unique presentation.

## Case presentation

A 57-year-old African-American male with a past medical history of chronic obstructive pulmonary disease, chronic alcoholism, and polysubstance abuse was admitted to the hospital with complaints of shortness of breath, weakness, and anuria for the last few days. He had been extremely depressed about losing his job and had been drinking heavily and snorting cocaine daily. He was not eating anything much and was relying heavily on these recreational drugs. The patient was taking intermittent bronchodilators. He was not on any statins. The vital signs revealed a temperature of 97.5 °F, blood pressure of 143/68 mmHg, heart rate of 78 bpm, respiratory rate of 16 per minute, and oxygen saturation of 98% on room air. Physical exam was remarkable for decreased skin turgor, dry mucous membrane, and bilateral wheeze on auscultation. The rest of the physical examination was unremarkable.

**Figure 1:**
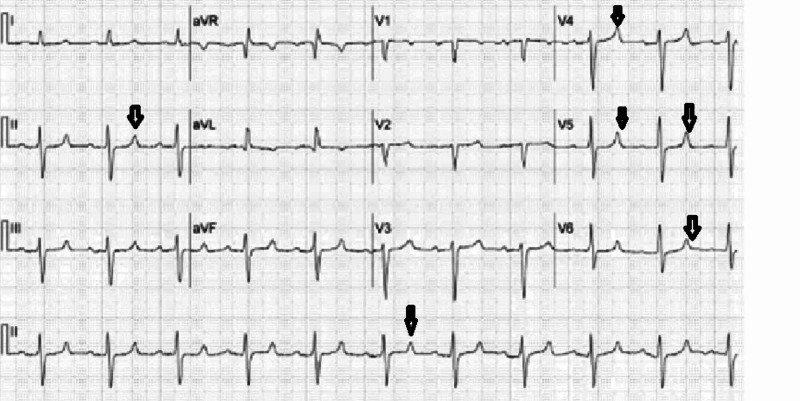
Electrocardiogram showing tall peaked T wave

**Figure 2:**
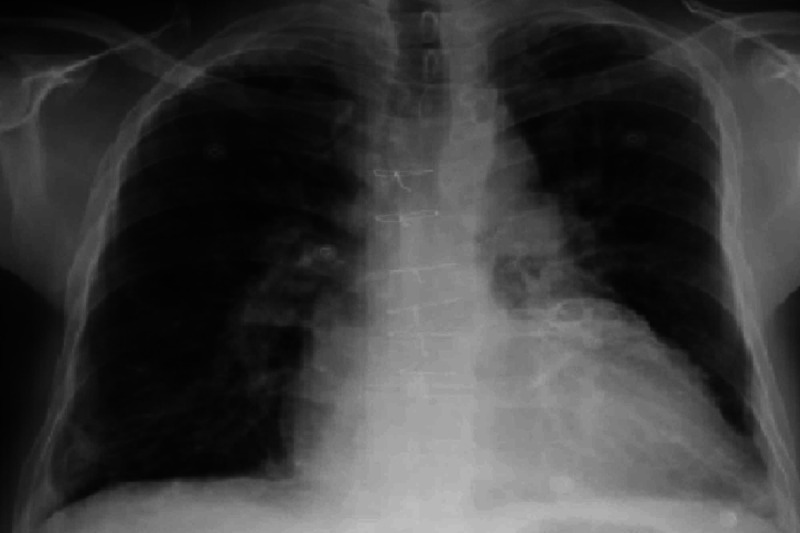
Chest X-Ray showing no acute abnormalities

Laboratory testing was done on admission day, which is summarized below (Tables 1, 2). Urine analysis was positive for blood, but no red blood cells were observed, and no casts were reported, which was likely secondary to myoglobinuria. Electrocardiogram (EKG) revealed a tall peaked T wave as shown in Figure 1. However, chest X-Ray was unremarkable, as shown in Figure 2.

**Table 1:** Summary of laboratory testing done on the day of admission BUN: blood urea nitrogen

Labs	Value	Reference
Serum sodium	124 mmol/L	135-145 mmol/L
Serum potassium	6.5 mmol/L	3.5-5 mmol/L
Serum calcium	5.5 mg/dl	8.5-10 mg/dl
Serum phosphorus	0.5 mg/dl	3-4.5 mg/dl
Serum CO2	20 mmol/L	23-30 mmol/L
Serum BUN	97 mg/dl	7-20 mg/dl
Serum creatinine	11 mg/dl	0.6-1.2 mg/dl
Serum albumin	3.6 g/dl	4-5.5 g/dl
Creatine kinase	141974 IU/L	22-198 IU/L
Troponin I	0.09 ng/ml	<0.04 ng/ml
Serum ethanol	<15 mg/dl	<15 mg/dl

**Table 2:** Results of the urine drug screen on the day of admission

Urine drug screen	Result	Reference
Amphetamine	Absent	Absent
Barbiturate	Absent	Absent
Benzodiazepine	Absent	Absent
Cocaine	Present	Absent
Marijuana	Absent	Absent
Methadone	Absent	Absent
Opiate	Absent	Absent
Phencyclidine	Absent	Absent

The patient was admitted to the intensive care unit for severe rhabdomyolysis with acute renal failure and massive electrolyte imbalance. He was treated aggressively with intravenous (IV) isotonic normal saline at 200 ml/hour; hypocalcemia was supplemented with IV calcium gluconate, and hyperkalemia was treated with oral sodium polystyrene sulfonate, IV calcium gluconate, insulin, and dextrose along with albuterol nebulization. He was also started on four-hourly beta-agonist nebulization along with IV steroids for his shortness of breath. Despite aggressive hydration and intense medical management, his serum potassium, and creatine kinase (CK) level remained elevated, and he was anuric throughout. Urgent hemodialysis (HD) was initiated for refractory hyperkalemia. After receiving HD for three consecutive days, his electrolyte imbalance improved while his CK started trending down. His clinical condition improved, and he was subsequently transferred to the floor. His CK level trend (IU/L) until discharge was as follows: 141974>164120>94833>96244>61464>9528>17432>8188>4514>3193>1672>552>108>58.

He continued to remain anuric despite his CK trending down; IV Lasix challenge and vigorous IV hydration did not help in renal recovery. He was switched to thrice-weekly intermittent HD. His blood urea nitrogen (BUN)/creatinine on the day of discharge was 32/3.4 mg/dl, and CK levels normalized to 58 IU/L. He continued to remain anuric and was discharged with a thrice-weekly outpatient intermittent HD scheduled.

Twelve months post-discharge, he continued to remain oliguric and was still was receiving regular thrice-weekly HD. He was strongly urged to get a renal biopsy, but he refused it every time, citing unclear reasons. He has been cocaine-free the whole duration but has continued with moderate alcohol consumption. He is in the process of being evaluated for a kidney transplant.

## Discussion

Cocaine is an illegal drug very frequently associated with visits to hospital emergency departments in the US. According to a 2011 report by a federal agency in the US, roughly 40.3% of illicit drug-related emergency department visits were related to cocaine only, compared to 36% for marijuana users 20.6% for heroin users [1].

Concomitant intake of ethanol and cocaine generates a pharmacologically active compound, cocaethylene. Cocaethylene formation contributes to longer-lasting and severe toxic effects of cocaine when used in conjunction with alcohol. Alcohol appears to potentiate cocaine hepatotoxicity in both humans and mice [3]. Cocaethylene has a long duration of action (based on the route of administration, it can be up to 13 hours) [4], and like cocaine, it is arrhythmogenic, while exerting vasoconstrictive, cardiac toxic, and neurotoxic effects [5]. Cocaethylene's physiologic effects far outlast the effects of cocaine alone administered by commonly used routes like inhalation or ingestion [4]. Levamisole is a common adulterant of cocaine that may lead to leukoencephalopathy, agranulocytosis, or cutaneous vasculitis. Levamisole-induced vasculitis and AKI have been reported in a few cases [6].

Cocaine use can impair kidney function through a variety of mechanisms [7]. A significant cause of AKI is cocaine-induced rhabdomyolysis [3]. O'Connor et al. showed that cocaine was the cause of severe rhabdomyolysis in 11% of patients among a pool of polysubstance abusers [8]. The decline in kidney function is observed in hypertensive patients using cocaine [9], and ESRD progression is accelerated from hypertensive nephrosclerosis [10]. Cocaine can also cause vasculitis and renal involvement through levamisole adulteration, as mentioned above [6].

Alcohol consumption is widely linked to cardiovascular and hepatic parenchyma injury. The link between alcohol use and kidney injury is controversial. The possible mechanism cited is oxidative stress leading to an excessive free radical generation, triggering increased inflammation and tissue injury [11]. No clear-cut association between alcohol-induced rhabdomyolysis and AKI has been reported. However, cocaethylene generation can potentiate vasoconstriction and AKI, as mentioned above [5].

Rhabdomyolysis came into widespread focus during the Second World War. However, historically it was first reported in 1881 from Germany [12]. Rhabdomyolysis is a syndrome constituting the release of intracellular muscle constituents into the circulation from muscle necrosis and breakdown. This includes intracellular metabolites (phosphate, urate, potassium) and intracellular proteins (aspartate transaminase, myoglobin, CK, lactate dehydrogenase, and aldolase) released into the bloodstream [13]. Common clinical features are myoglobinuria and muscle pain, while CK levels are elevated in labs. The spectrum of the disease varied from asymptomatic serum muscle enzymes elevation to life-threatening disease characterized by extreme enzyme elevations, electrolyte derangement, with profound AKI. The reported frequency of AKI ranges from 15 to over 50% [14,15]. The variability of AKI incidence is probably linked to the severity of underlying rhabdomyolysis and AKI definition inconsistencies [14]. CK levels of lower than 15,000 to 20,000 IU/L at admission have a relatively decreased risk of progressing to AKI; risk factors in such patients being sepsis, volume depletion, and acidosis [16]. Pathogenesis of renal dysfunction in the usual presentation of rhabdomyolysis includes heme pigment casts-induced renal tubular obstruction, volume depletion-induced renal ischemia, vasoconstriction-induced reduced blood flow to the outer medulla, and free chelatable iron-induced tubular destruction.

Traditionally, obese patients, male individuals, African Americans, and patients younger than 10 years of age and older than 60 years all have a higher incidence of rhabdomyolysis. In adults, rhabdomyolysis is most commonly multifactorial, frequently involving trauma and illicit drugs. AKI plays a significant role in mortality from rhabdomyolysis. A study published by McMahon et al. in 2013 helped to establish the risk prediction score of AKI in rhabdomyolysis based on CK levels and other parameters [17]. With muscle injury, CK is released from injured muscle into the circulation, resulting in a dramatic (sometimes over 1,000-fold) increase in its serum concentration. Levels generally reach a maximum within 24 hours of a focal muscle injury and, after that, decline by 50% every 48 hours [18]. AKI is uncommon when peak CK levels are under 15,000 IU/L [16]. In the study by McMahon et al., the two cohorts of 2,371 rhabdomyolysis patients failed to demonstrate a linear relationship between CK concentration and composite consequence of severe AKI or death [17]. However, CK levels of >40,000 IU/L was accompanied by increased overall risk in unadjusted logistic regression models. Treatment of rhabdomyolysis mainly consists of aggressive volume resuscitation, urine alkalization and diuresis, and HD when necessary for rhabdomyolysis-induced AKI [19].

In our case, the patent's prolonged kidney failure and ultimate ESRD could be attributed to the severe rhabdomyolysis-induced acute renal toxicity and the inability to achieve renal recovery from likely further episodes of repeated undiagnosed rhabdomyolysis from alcohol ingestion given his history of depression. Though kidney biopsy would have given definitive results, cocaine and alcohol co-ingestion played a significant role in his ESRD.

## Conclusions

Cocaine intoxication with alcohol co-ingestion is a lethal combination that can lead to severe rhabdomyolysis and even AKI, often leading to a condition where HD is required. Sometimes, the association can cause renal damage severe enough to culminate in ESRD. This association needs to be treated aggressively as the chances of severe AKI are high. Patients need to be educated and cautioned about this possible severe adverse effect of alcohol and cocaine co-ingestion. Further studies on cocaethylene-induced nephrotoxicity need to be conducted to gain more insights into this condition.
